# Traumatic Surgical Conditions of the Bladder, and Treatment

**Published:** 1900-06

**Authors:** W. A. Duringer

**Affiliations:** Fort Worth, Texas


For Texas Medical Journal.

### Traumatic Surgical Conditions of the Bladder, and Treatment.

BY W. A. DURINGER, M. D., FORT WORTH, TEXAS.

First in frequency we will mention those lesions of the bladder due to rough and injudicious use of instruments and the presence of foreign bodies. The average young physician deems it essential and extremely important to introduce the sound or other instruments into the bladder, whereby he can demonstrate his skill to the patient and ignorance to the profession. More especially is this noticeable or likely to occur when there is an already existing cystitis or the presence of a foreign body.

Second in frequency, we have accidents to this viscus during surgical operations for other pathological conditions of the pelvic cavity. The reason for this frequent occurrence is obvious; almost any of the laparotomies performed at this time for malignant growths, tumors, inflammations, adhesions, etc., involving the pelvic viscera to any great extent a few years ago were considered inoperable, and are now being done by bold, and I might say irresponsible, surgeons, who consider reputation as a cutter more than human life.

We will place next in frequency rupture of the bladder, which usually occurs in an already diseased bladder, where the walls have become weakened by previous disease, comatose conditions where we are called and find the patient's bladder distended to the umbilicus, or during a drunken debauch when the patient's bladder is usually filled, and, while in this condition, he participates in a street brawl and receives a kick in the hypogastric region, falls or is thrown down stairs, either of which is quite sufficient to produce rupture of a sound bladder.

The next frequency is stabs and gunshot wounds. It seems almost impossible to appreciate a stab in the bladder when empty, but this organ is not always in a state of collapse, and especially when such accidents are liable to occur.

That they do occur is nevertheless a fact, and usually accompanied by extensive extravasation of urine both extra and intra-peritoneal. Gunshot wound of bladder is so seldom encountered by the surgeon that its rarity makes it all the more interesting. They have always been regarded as fatal wounds; this fatality accounted for probably by damage done to other important abdominal viscera by the ball in its course through the body; but usually death occurs as in other varieties of injuries mentioned above, from extravasation and hemorrhage.

### SYMPTOMS AND DIAGNOSIS.

Symptoms of the first variety, due to instrumentation, foreign bodies, etc., are simply an exaggeration of cystitis and sometimes hemorrhage. They should not be considered too lightly, for, indeed, many times it proves the match which lights the fire of destruction.

An abrasion, destruction of a patch of mucous membrane too often proves a focus of infection resulting in interstitial abscesses, necrosis, gangrene and death.

Symptoms of the second variety, viz.: laparotomies, are usually manifest at the time of operation, and if repaired properly all will be well, but, in many instances, they are first discovered in the dead-house. Twenty-four or forty-eight hours after the operation patient dies of collapse and shock; the kidneys have failed to secrete any urine and the patient presumably died of urina. Post-mortem reveals the true cause—*neglect of bladder wound.*

Symptoms of the third variety, viz.: rupture, are usually well marked. In a few hours the once distended bladder is no more, and, if the patient is rational, he is conscious that something has suddenly given way, and for the time is completely relieved.

Catheter introduced into the bladder reveals the absence of urine. No alarming symptoms yet developed except, probably, tenesmus, which we are too apt to attribute to the effect of the debauch upon the urine or an already diseased bladder spoken of. The careful surgeon will now investigate further, and inject the bladder with a given quantity of fluid, viz., Tierch or normal saline solution or one of boracic acid, and determine its ability to retain this given quantity. If a leakage occur it can be determined by the quantity of fluid returned through the catheter. Allow this fluid to remain in the bladder for a while, for the rent may be small, leakage slow, and the examiner deceived. The usual symptoms accompanying rupture will soon be manifest: collapse, prostration, hiccough, and an inability to void urine by the natural channel.

Fourth variety: I have included stabs and gunshot wounds under the same head, for the symptoms are the same; that is, tenesmus, blood, and only a small quantity of urine voided. The bladder is in a state of tonic contraction, wound partially closed, leakage or extravasation slow; alarming symptoms slow as a consequence.

Extravasation and infectious inflammation is sure to occur, leading to gangrene, septicæmia and death.

Another condition suggests itself here: that is, gunshot wounds involving both bowels and bladder; the immediate symptoms are the same as already described, with the addition of a greater degree of shock, distention, extreme tenderness over abdomen, and rigidity of abdominal muscles.

TREATMENT.

Of the first variety, instrumentation, foreign bodies, etc., if properly administered, responds promptly and effectually. If a foreign body be present, remove it after one of the recognized surgical methods adapted to such work. If an abrasion or loss of structure without perforation has been produced, place the bladder at rest with the usual sedatives, and keep its contents as aseptic as possible; this is also applicable after the removal of foreign bodies.

Treatment of the second variety, accompanying laparotomies, if recognized at the time of operation and only torn or incised it must be closed by stitches, chromatized catgut, small silk or flax; mucus and muscular layer approximated. If a portion of the body of the bladder is destroyed or resected, its walls must be properly approximated and leakage guarded against. The bladder must be kept in a contracted state to prevent tention on stitches and leakage; this is accomplished by suprapubic or perineal drainage: suprapubic by twisted roll of gauze, wick fashion, down to injured point of the bladder; perineal by perineal section, introducing tube in the bladder. You then have an ideal drainage.

This I much prefer to the retention of catheter in the urethra; reasons are obvious.

*Treatment for Ruptured Bladder.*—If we were positive that the extravasation was extra-peritoneal our procedure would be clear; this we do not know nor can we determine, especially for some time, without resorting to operative measures, which should be resorted to in all cases as soon as the diagnosis is made.

If fortune has smiled upon the patient and we see evidences of extravasation extra-peritoneal only, then we proceed at once through the space of Retzius, search for rent in bladder and drain. We will be assisted in this procedure by injecting the bladder and following the course of leakage; if drainage is not complete by this route drain by the perineum also, and keep the bladder empty, thus allowing the rent to heal. If the extravasation has found its way up the ilium and in the lumbar region it will then be necessary to make a counter-opening and drain here also, never forgetting the importance of free drainage. If the character of rupture, extra or intra-peritoneal, cannot be determined at once, then it becomes your duty to open the peritoneum and investigate. If the peritoneum has been ruptured stitch it, wash out and close the abdomen; then work extra-peritoneally, repair damage to bladder, and if you think best drain as in the preceding.

Fourth. In gunshot wounds of the bladder the same rule applies, for we are never certain that the ball has not penetrated the peritoneal cavity, hence the importance of a laparotomy in all such injuries, and if the bowels have been wounded, artery severed, accompanied by a profuse hemorrhage, we are then in a condition to repair the damage done in the abdominal cavity. After that has been attended to properly, wash out, close the peritoneal sack and finish our work extra-peritoneally upon the bladder. This can be cared for as in the preceding cases by dissecting off the peritoneum to point of wound, closing it by sutures or leaving wound open and draining, keeping bladder empty; the wound in the bladder heals readily without stitching.

In keeping with the above facts, I will report a case that occurred in my own practice, since I determined to bring this subject before you.

J. I., age about 37, railroad conductor, received gunshot wound of left inguinal region, 41-calibre ball, three weeks ago. Found patient sitting in chair and voiding but a few drops of urine and blood every few minutes. The vesical tenesmus was intense, abdominal muscles hard, contracted and tender. Unable to lie down on account of extreme pain in abdomen. Was given hypodermic of one-half grain of morphia, and his condition explained to him. He was told that his wound was mortal, and laparotomy suggested, with the hopes of saving his life. This he readily consented to, and was immediately removed to the infirmary in ambulance. In three hours from receipt of injury laparotomy in the median line was done. Wound in bladder, posterior, just above trigone, was found and peritoneal opening at this point stitched with flax. Then I proceeded to look for injury done abdominal viscera, and was soon rewarded by finding four bullet holes, each admitting the end of the finger, in a coil of the small intestines.

The surface of wounds were bathed with bichloride and mucous and muscular surfaces approximated and Zermy stitch over all to give adhesive surface and support; flax, or linen thread used here also.

Abdomen washed thoroughly with sterile water and another search instituted; no further damage being found the peritoneal cavity was closed with catgut. The bladder next claimed my attention, and, with the assistance of bogie in bladder, it was elevated and peritoneum stripped from fundus and posterior surface until bullet hole was reached. Soft catheter was inserted through wound into bladder and left for drainage. Retzius space was washed thoroughly and strips of iodoform gauze packed around catheter and fundus of bladder.

Abdominal wound now closed with silkworm gut down to suprapubic space, which was left open for drainage, the same as in suprapubic cystotomy. Hypodermic of morphia given every four or six hours for two days, and then five grs. quin. s., one gr. opii., one gr. of tr. of aconite given every four or six hours. You will see that this treatment is a little contrary to that usually following laparotomies, but my object was to "splint" the bowels and give these sutured holes a chance to heal.

Temperature or pulse never went above 100° during the entire time. At the end of four days the tube was removed from bladder and twisted gauze carried well down in wound, was substituted; patient instructed to void his urine naturally every hour. After the fifth day no more leakage by the supra-pubic route. About the seventh day patient complained of severe pain in bowels; no fever and no symptoms of peritonitis. I concluded they were gas pains, and gave him one pint of sweet oil by enema and one grain of calomel and soda every hour until six doses were taken. This succeeded in moving his bowels freely, as well as pain. Since then he has made an uninterrupted recovery. Bowels moving regularly and urine passing by the natural channel.

